# Can digital self-screening improve identification of chronic dyspnoea in Australian general practice? A proof-of-concept protocol for the BREATHE SMART trial

**DOI:** 10.1136/bmjopen-2025-110702

**Published:** 2026-05-29

**Authors:** Kyumin Jang, Katrina Giskes, Allison Martin, Anthony Paulo Sunjaya, Phyu Khin, Christine Jenkins, Charlotte Mary Hespe, on behalf of the BREATHE SMART Steering Committee, Charlotte Hespe

**Affiliations:** 1 Department of General Practice and Primary Care Research, The University of Notre Dame Australia School of Medicine, Darlinghurst, New South Wales, Australia; 2 The George Institute for Global Health, Sydney, New South Wales, Australia; 3 Faculty of Medicine, University of New South Wales, Sydney, New South Wales, Australia

**Keywords:** Primary Care, RESPIRATORY MEDICINE (see Thoracic Medicine), Digital Technology

## Abstract

**Introduction:**

Chronic dyspnoea is a prevalent and clinically significant symptom, often indicative of underlying cardiorespiratory disease. It is frequently under-reported by patients and under-recognised in primary care, with these challenges exacerbated in rural and remote communities where disease burden is greater and patients experience barriers to timely diagnosis and management. The BREATHE SMART trial aims to implement and evaluate an innovative, fully digital self-screening system for chronic dyspnoea, integrated into general practice workflows and information technology infrastructure. This approach seeks to enhance early detection and management of chronic cardiorespiratory conditions across diverse practice settings.

**Methods and analysis:**

This multisite proof-of-concept study will test a software platform delivering a preconsultation self-screening questionnaire across 40 general practices in urban, rural and remote Australia. The system identifies eligible patients (≥18 years, consenting to SMS communication with their practice), issues an automated SMS that administers a validated dyspnoea screening questionnaire, and summarises responses for integration into the electronic medical record. Process evaluation will assess acceptability and utility using deidentified audit data, software metrics and qualitative feedback from patients, staff and general practitioners (GPs) via surveys, interviews and focus groups. Approximately 12 000 patients will be screened over 12 months. Primary outcomes will include the proportion completing self-screening and prevalence of chronic dyspnoea and secondary outcomes will include the rate of newly diagnosed chronic dyspnoea-related conditions (ie, asthma, chronic obstructive pulmonary disease and heart failure) in the preceding 12 months and during the intervention period.

**Ethics and dissemination:**

Ethics approval was granted by the University of New South Wales Human Research Ethics Committee (HREC) (iRECS6645) and the University of Notre Dame Australia HREC (2024-155). Participating practices and each GP will provide written, informed consent. All patients being screened will provide electronic informed consent. Results of the study will be disseminated through various forums, including peer-reviewed publications and presentation at national and international conferences. Following the study, participating practices will be provided with a summary of the findings of the study, together with a full copy of any publications and a plain language statement for participants, which will be made available in the practices.

**Trial registration number:**

ACTRN12624001451594.

**Strengths and limitations of this study**

- BREATHE SMART is a proof-of-concept study that implements a fully integrated, digital self-screening system for chronic dyspnoea in general practice that aligns with existing workflows and IT systems.
- BREATHE SMART will identify and address the burden of unreported or undetected dyspnoea in the community, thereby improving patient outcomes and strengthening the preventive health capacity of general practitioners.
- BREATHE SMART includes practices across urban, rural and remote areas, enabling evaluation of equity, feasibility and scalability in diverse settings.
- As a proof-of-concept study, generalisability may be limited until tested at scale.
- Reliance on digital communication and self-reporting may exclude individuals with lower health or digital literacy, potentially affecting reach and equity.

## Introduction

 Chronic breathlessness (dyspnoea) affects over 1.8 million Australians and significantly limits physical activity.^1^ It is a common symptom in primary care, experienced by approximately 10% of the population.^1^ Dyspnoea is multifactorial—often linked to cardiorespiratory disease, obesity, deconditioning, anxiety or a combination of these—and is frequently under-recognised and under-reported by patients. In older adults, dyspnoea is often attributed to ageing, reduced physical activity, or other comorbidities.^2^ The underlying causes of dyspnoea vary substantially across age groups,^3^ with distinct diagnostic approaches required depending on whether the aetiology is cardiac, respiratory, or otherwise,^4^ highlighting the complexity of recognising, investigating, and treating this common symptom.

The burden of chronic dyspnoea is often exacerbated or caused by concurrent medical or environmental conditions. For instance, obesity increases the likelihood of experiencing significant breathlessness fourfold and is becoming increasingly prevalent across all age groups.^5^ Environmental factors, such as the 2019–2020 bushfires, along with the ongoing impacts of SARS-CoV-2, have further contributed to rising rates of breathlessness in the community.^6 7^ A national survey of over 10 000 Australian adults found that 22% reported a current respiratory or cardiac condition.^1^

General practice remains the most frequent point of contact within the Australian health system, with over 85% of the population consulting a General Practitioner (GP) at least once a year.^2^ This positions GPs as key to the screening and management of chronic dyspnoea. However, GPs face challenges in identifying dyspnoea, due to its complex and multifactorial causes,^8 9^ and it is often only detected during acute presentations^10^; additionally, increased patient complexity, workforce shortages and funding that prioritises acute care over prevention^11^ are barriers for preventive care in general practice. Improving the detection of chronic dyspnoea is important, given the condition’s high prevalence and associated morbidity.^1 6 12 13^

One promising strategy to improve early detection is to better use the time prior to a patient’s GP consultation as a moment in patient care. Our group has demonstrated that digital self-screening—conducted via waiting room kiosks or smartphones—can be successfully integrated into general practice and can improve case detection. This model has previously increased the diagnosis of asymptomatic cardiac arrhythmias^14^ and familial hypercholesterolaemia.^15^ However, many digital tools fail to deliver meaningful impact because they disrupt the natural flow of care, so for an innovation in general practice to be effective, it must fit into existing systems without adding to time pressures or increasing staff workload.^16^ The BREATHE SMART study addresses this challenge by testing a preconsultation screening tool for chronic dyspnoea, designed to improve the identification of undiagnosed chronic breathlessness in primary care.

## Methods

The study will recruit patients aged 18 years and older who have consented to SMS communication with their practice who are scheduled for a GP consultation, either face-to-face or via telehealth. Participating practices must use Best Practice electronic medical record (eMR) software. Recruitment commenced in February 2025 and is planned to cease in December 2026.

### Study design

BREATHE SMART is an interventional study to be implemented across 40 general practices in urban, regional and rural areas of Australia. The study will be in parallel with the BREATHE CDSS study, which is an open-label, cluster-randomised controlled trial comparing standard care with a clinical decision support system (CDSS) intervention.^17^ Practices will be randomised to the CDSS at the practice level using a computer-generated allocation sequence prepared by an independent statistician who is not involved in recruitment or data collection. Allocation will occur only after a practice has formally consented to participate, ensuring concealment of assignment during the recruitment phase.

BREATHE SMART and BREATHE CDSS are related but methodologically separate components of our broader programme to improve the identification and management of chronic dyspnoea in general practice. BREATHE SMART is a proof-of-concept study evaluating the feasibility and acceptability of a digital self-screening pathway completed before the GP consultation. BREATHE CDSS is a separate study focused on developing and testing a CDSS to guide GPs once a patient with potential chronic breathlessness has been identified.^17^ They form a complementary sequence: BREATHE SMART supports earlier identification, while the BREATHE CDSS supports structured diagnostic decision-making.

### Study outcomes

The primary outcome measures of BREATHE SMART are:

- The proportion of eligible patients who complete opportunistic self-screening for chronic dyspnoea.
- The proportion of screened patients identified as having chronic dyspnoea.

The secondary outcome measures are:

- The incidence of dyspnoea and related conditions (ie, chronic obstructive pulmonary disease (COPD), asthma and heart failure) per 1000 patients in general practices before and after implementation of the BREATHE SMART intervention.

In addition to these outcomes, the study will also examine:

- The acceptability, competing demands, barriers and facilitators of the self-screening process from the perspectives of practice staff and patients.
- GPs’ acceptance of, engagement with and perceived utility of the digital dyspnoea screening and management system.

### Customised integration software

Customised integration software has been developed for the BREATHE SMART study to automate the self-screening process and embed it within existing general practice workflows. This development will be undertaken in collaboration with our software partners, BetterConsult and Best Practice, ensuring compatibility with current systems and minimising disruption to routine clinical operations.

**Figure 1:**
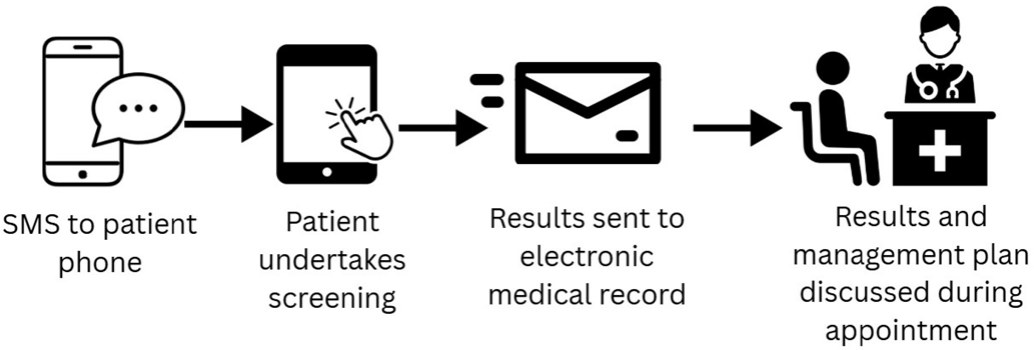
Integration of dyspnoea self-screening with GP consultation workflow. GP, general practitioner.

The patient flow for the BREATHE SMART study is summarised in figure 1. Prior to their GP appointment, patients receive an SMS invitation prompting them to complete a brief screening questionnaire on their smartphone. This digital screening is designed to identify symptoms of breathlessness, using the modified Medical Research Council (mMRC) Dyspnoea Scale (table 1).

**Table 1:** The modified Medical Research Council (mMRC) scale

mMRC grade	Description
0	Not troubled by breathlessness except on strenuous exercise
1	Troubled by shortness of breath when hurrying on the level or walking up a slight hill
2	Walks slower than people of the same age on the level because of breathlessness or has to stop for breath when walking at own pace
3	Stops for breath after walking about 100 m or after a few minutes on the level
4	Too breathless to leave the house or breathless when dressing or undressing

mMRC, modified Medical Research Council.

Once completed, the patient’s responses are automatically transmitted to their eMR. During the consultation, the GP reviews the screening results and, where appropriate, initiates a discussion regarding potential investigations, causes and management strategies for breathlessness.

### Measurement of chronic dyspnoea

The mMRC (table 1) is a validated, single-item tool used to assess the degree of functional impairment due to breathlessness.^9^ It grades dyspnoea on a scale from 0 to 4 based on the level of physical activity that provokes symptoms, ranging from no breathlessness except with strenuous exercise (grade 0) to breathlessness that prevents the patient from leaving the house or occurs during basic activities such as dressing (grade 4).^18 19^ The mMRC scale is widely used in clinical practice and research to classify symptom severity, particularly in patients with chronic respiratory diseases such as COPD, and has demonstrated strong correlations with morbidity, mortality and quality of life.^20 21^

Patients who report an mMRC score of 0—indicating breathlessness only with strenuous exercise—will not proceed further in the screening pathway. All patients reporting an mMRC≥1 will be asked to indicate how long they have experienced this level of breathlessness. Those reporting symptoms for 4 weeks or more will be classified as experiencing ‘chronic dyspnoea’ and flagged for further clinical assessment.

All patients identified as experiencing chronic dyspnoea will be invited to complete the Dyspnoea-12 (D-12) questionnaire.^22^ The D-12 is a validated instrument designed to assess the severity and multidimensional nature of breathlessness, incorporating both physical and emotional components.^22^ It has demonstrated strong psychometric properties across diverse patient populations, making it a reliable tool for clinical and research settings.^22 23^

### Practice recruitment

40 general practices will be recruited from urban, regional and remote areas across Australia, with data collection occurring over a 12-month period at each site. Recruitment will leverage established partnerships with key organisations, including BetterConsult, the Royal Australian College of General Practitioners, the Rural Doctors Network, Primary Health Networks and corporate medical centre groups.

To reduce the potential for recruitment bias at the practice level, we will use a standardised recruitment approach. All practices will receive the same study information, and recruitment to the study will not be targeted based on practice performance, digital maturity or patient demographics. We will document reasons for non-participation where provided, enabling transparent reporting of recruitment flow and potential sources of bias.

### Consents

This study involves three levels of consent (refer to online supplemental materials) for consent forms.

#### General practice and practitioner consent

Written informed consent will be obtained from each participating general practice, including all participating GPs. This consent covers the use of patient responses to the survey for research purposes, as well as the installation and use of the BetterConsult digital tool to facilitate the intervention.

#### Patient consent for self-screening

Eligible patients who have previously consented to receive SMS communications from their general practice will receive a text message inviting them to complete the BetterConsult questionnaire prior to their GP consultation. By accessing the questionnaire via the provided link, patients are presented with information about the screening tool and its purpose. Completion of the questionnaire will be taken as an indication of consent to participate in the BREATHE SMART study.

#### Consent for process evaluation interviews

Separate written informed consent will be obtained from all practice staff and patients who agree to participate in qualitative interviews as part of the process evaluation. Interviews will be conducted in private settings to ensure confidentiality, and participants may withdraw or terminate the interview at any time without consequence, should any discomfort arise.

### Training and support

The research team will deliver tailored training to GPs participating in the trial. Training sessions will be scheduled at times convenient for practice staff and will include approximately 30 min with GPs. Supplementary training materials, including on-demand videos and laminated quick reference guides will also be provided. Ongoing support will be offered throughout the study, including remote assistance with GP onboarding, troubleshooting, addressing queries.

### Remuneration

Participating general practices will receive an upfront payment of $A1000 to compensate for administrative tasks, data entry and time spent on patient recruitment and consent. Intervention sites will receive an additional $A500 for enrolling 1–99 patients, with further payments of $A500 for each subsequent 100 patients, up to a maximum of $A4000 per practice. These payments are intended to offset the administrative and follow-up workload associated with participation in the implementation research project.

### Process evaluation

The process evaluation will examine the end-user experience of the BREATHE SMART intervention. Specifically, it will examine the feasibility and acceptability and identify contextual factors (barriers and enablers) that influence participation in dyspnoea self-screening according to patients, practice staff and GPs. The process evaluation will comprise three components: platform performance metrics, a process evaluation questionnaire and process evaluation interviews- each of these is described below.

#### Platform performance metrics

A number of platform metrics will be used to assess different aspects of software performance and user engagement. These domains are summarised in table 2.

**Table 2:** Platform metrics that will be used in the process evaluation

Metric domains	Assessment
Interaction metrics	How long patients interact with the screening software How long GPs interact with the screening results
Adoption and reach	Proportion of in-scope patients that interact with the screening software and proportion who complete self-screening
Usage patterns	GPs’ usage of the screening results
Clinical impact	In 12 months of intervention compared with 12 months prior to intervention: Diagnosis of dyspnoea-related conditions Investigations (imaging, spirometry, bloods) for patients with dyspnoea Prescribing of medications for dyspnoea-related conditions (ie, asthma, COPD, heart failure) intervention Acute presentations of conditions related to dyspnoea (exacerbations of asthma, COPD, CCF)

CCF, congestive cardiac failure; COPD, chronic obstructive pulmonary disease; GP, general practitioner.

- Reach assesses how broadly the intervention is accessed across different patient demographics and practice settings.
- Adoption refers to the proportion of the target population that begins using the software. High adoption rates indicate that the software is perceived as valuable and accessible.
- Interaction metrics track how often and for how long users interact with the software, providing insights into usability. High levels of engagement typically suggest that the platform is user-friendly and meets the needs of its intended audience, whereas initial high engagement that rapidly declines suggests engagement was not maintained. Measures of adoption and reach can also be used to assess platform performance.
- Usage patterns analyse patterns of software use- including which aspects of the software are effective, which information is most useful to users and which components may require redesign or further development.
- Linking user engagement data with health outcomes, the study will assess whether higher engagement leads to improved health outcomes.

#### Process evaluation questionnaires

At least 2 months after the commencement of the trial, process evaluation questionnaires will be distributed to participating patients, GPs and practice staff. The aim is to identify both system-level and individual-level factors that may influence the broader implementation and scalability of the screening programme. The evaluation will assess the acceptability, usability and sustainability of the screening process from the perspectives of these key stakeholders.

#### Process evaluation interviews

Semistructured interviews will be conducted with patients, GPs and practice staff as part of the detailed process evaluation. All interviews will be audio-recorded, transcribed verbatim and analysed using thematic analysis. Both inductive (data-driven) and deductive (theory-informed) approaches will be applied to identify recurrent themes and patterns, guided by the core evaluation questions. Thematic coding generation will continue until saturation is reached.

Patient interviews will explore satisfaction with the self-screening process, including perceptions of the SMS prompts, ease of use and any barriers or facilitators encountered. Interviews with practice staff will focus on the acceptability of the self-screening approach, its integration into routine workflows and any challenges or enablers experienced. Interviews with GPs will explore their experience with the intervention- from screening to clinical management- including its impact on consultation flow, practice efficiency and recommendations for future scale-up.

### Data extraction

eMR software will be utilised in each participating practice to collect relevant deidentified data from the practice database. This will include the total number of in-scope patients with a GP appointment over the study period as well as the incidence of chronic dyspnoea and related conditions- including COPD, asthma and heart failure- during the 12 months before and the 12 months following implementation of the BREATHE SMART intervention.

### Statistical analyses

Descriptive analyses will be conducted at both the individual practice level and across all practices combined.

Preintervention data analysis will include:

- Total number of in-scope patients actively attending the practice;
- Total number of patients newly identified with dyspnoea and a range of conditions related to dyspnoea (eg, asthma, COPD, heart failure) in the preintervention period;
- The rate of detection (per 1000 patients) of dyspnoea and dyspnoea-related conditions;

Intervention period data analyses will include:

- Total number of in-scope patients actively attending the practice over the study period;
- Total number of in-scope patients completing dyspnoea self-screening;
- Total number of patients identified with dyspnoea (ie, mMRC≥1 requiring further investigation;
- The detection rate of dyspnoea requiring further investigation (per 1000 patients);
- The detection rate of newly-detected conditions related to dyspnoea (per 1000 patients) (eg, asthma, COPD, heart failure).

## Ethics and dissemination

The study protocol (version 3.0 23 August 2024) has been approved by the University of New South Wales (UNSW) Human Research Ethics Committee (HREC) (iRECS6645) and the protocol for the process evaluation study has been approved by the University of Notre Dame Australia (UNDA) HREC (2024-155). These comply with the National Health and Medical Research Council’s ethical guidelines. All protocol amendments will be submitted and approved through the UNSW and UNDA HRECs, and sent to all participating GP practices. BREATHE SMART has been registered on the Australian and New Zealand Clinical Trial Registry (ACTRN12624001455550, registered 16 December 2024, (https://anzctr.org.au/Trial/Registration/TrialReview.aspx?id=388712). Details for accessing the trial protocol can be found at: https://anzctr.org.au/Trial/Registration/TrialReview.aspx?id=388712. Results of the study will be disseminated through various forums, including peer reviewed publications and presentation at national and international conferences. Authorship of publications will follow International Committee of Medical Journal Editors guidelines and the BREATHE publication policy, and no professional writers will be employed nor artificial intelligence used. Following the study, participating practices will be provided with a summary of the findings of the study, together with a full copy of any publications and a plain language statement for participants, which will be made available in the practice reception area.

## Discussion

Chronic breathlessness is a common and debilitating symptom, affecting up to one in ten adults in the general population.^1 8^ It is often under-recognised in primary care, yet serves as an early warning sign for underlying conditions, including cardiorespiratory disease.^1 8 13^ Early identification and management of chronic breathlessness can lead to improved patient outcomes and reduced healthcare burden.^24^ However, the episodic and time-constrained nature of general practice consultations is a barrier to the routine identification of breathlessness. The use of a patient-facing, self-screening breathlessness item incorporated into routine preappointment questions may offer a practical and scalable solution to address this gap.

Digital self-screening tools have been explored in general practice primarily for chronic disease risk factor screening.^15^ While these tools have demonstrated feasibility and acceptability,^25^ their application to symptom-based screening, such as for breathlessness, remains under-investigated. Studies suggest that both patients and GPs generally perceive pre-consultation self-screening as acceptable and beneficial, particularly when the process is automated and embedded into everyday clinical practice.^14 15^

There are a number of limitations of the study design. The operationalisation of GP engagement with screening results—using the number of dyspnoea related conditions recorded at each site—captures only one dimension of how GPs may use the information. This approach does not allow us to observe the full breadth of clinical reasoning, nor does it capture instances where GPs may discuss breathlessness but not record a specific diagnostic code. As such, some nuances of GP–patient interaction and decision making may be missed. This measure is intended as a pragmatic proxy for assessing whether the screening information prompts diagnostic consideration, rather than a comprehensive assessment of clinical management.

Furthermore, only patients with a mobile phone who have opted in to receive SMS communications will be able to participate in the digital screening pathway. While this design choice reflects real world implementation constraints and the current capabilities of the digital platform, it introduces limitations related to digital access, digital literacy, and patient preference. These factors may lead to under representation of certain groups, including older adults, people with lower socioeconomic status, and those with limited English proficiency. We note that this may affect estimates of reach and uptake and should be considered when interpreting feasibility outcomes.

In addition to the above-mentioned factors, several challenges persist in achieving widespread implementation of digital self-screening tools in general practice. High staff workload, disruptions to clinical workflow, and delays between screening and clinical decision-making have hindered the integration of similar interventions in routine care.^26 27^ The BREATHE SMART study evaluates a fully automated, patient-operated breathlessness self-screening deployed prior to GP consultations. This approach is specifically designed to minimise disruption and reduce the time burden on clinical staff. By addressing known barriers to implementation, the system aims to support earlier detection of chronic breathlessness in primary care settings.

By automating symptom capture and integrating it into routine consultations, the BREATHE SMART model has the potential to improve the identification of breathlessness in general practice. If demonstrated to be feasible and acceptable, this approach may offer a scalable strategy for implementing guideline-based breathlessness care. In doing so, it could help reduce diagnostic delays and improve health outcomes for patients with undiagnosed or under-treated cardiorespiratory conditions, especially among patients in regional and rural/remote areas, and the screening results could then inform a clinical tool that provides GPs with evidence-based recommendations to guide diagnostic and management pathways.

## Supplementary material

10.1136/bmjopen-2025-110702 online supplemental file 1
