# Supplementary material for: Can digital self-screening improve identification of chronic dyspnoea in Australian general practice? A proof-of-concept protocol for the BREATHE SMART trial
Source: BMJ Open. 2026 May 29;16(5):e110702. doi: 10.1136/bmjopen-2025-110702 (PMC13223636; doi:10.1136/bmjopen-2025-110702)
Supplement: Supplementary file 2 [file bmjopen-2025-110702.reviewer_comments.pdf]

PEER REVIEW HISTORY

BMJ Open publishes all reviews undertaken for accepted manuscripts. Reviewers are asked to complete a checklist review form and are provided with free text boxes to elaborate on their assessment. These free text comments are reproduced below.

ARTICLE DETAILS

Title (Provisional)

Can digital self-screening improve identification of chronic dyspnoea in Australian general practice? A proof-of-concept protocol for the BREATHE SMART trial

Authors

Jang, Kyumin; Giskes, Katrina; Martin, Allison; Sunjaya, Anthony Paulo; Khin, Phyu; Jenkins, Christine; Hespe, Charlotte Mary; Steering Committee, BREATHE SMART

VERSION 1 - REVIEW

**Reviewer**	**1**
**Name**	**Lo, Stephen B**
**Affiliation**	**Ohio State University Health System**
**Date**	**27-Feb-2026**
**COI**	**None**

Thank you for the opportunity to review this manuscript, a protocol paper of a multi-site proof-of-concept study to implement and evaluate a digital self-screening questionnaire to assess dyspnea across Australia. This is an important effort given the prevalence, clinical significance, yet under-recognition of dyspnea. The protocol is well thought out and has the potential to fill an important supportive care need. Below are my comments:

• Additional detail on how BREATH SMART and BREATHE CDSS will overlap as these studies are running in parallel

• Including clarity on the eligibility criteria of who would be considered an eligible patient to include in the denominator would be useful. Would this be all-comers regardless of the ability to engage in screening due to language, visual deficits, etc.?

• Although this study is proof of concept, it would be useful to identify the primary study outcome and any a priori definitions of how you would operationalize “success” for each outcome (e.g., what would be considered a successful proportion of eligible patients screened?)

• Details on the randomization process would be useful. Any stratification of practices? Any additional details on how practices will be recruited to reduce bias?

• Addressing some limitations of the design could be useful in the discussion. For example, the study appears to be operationalizing GPs' discussion of dyspnea screening results and management based on the number of dyspnea-related conditions reported at a site. Commenting on what could be missed with this operationalization could help provide further context on what this protocol can address or not. Similarly, only patients with a phone that opted in to receive SMS communications will be screened. This design choice, though absolutely understandable, has its own limitations that can be mentioned.

VERSION 1 - AUTHOR RESPONSE

Responses to Reviewer's comments

1. Thank you for the opportunity to review this manuscript, a protocol paper of a multi-site proof-of-concept study to implement and evaluate a digital self-screening questionnaire to assess dyspnea across Australia. This is an important effort given the prevalence, clinical significance, yet under-recognition of dyspnea. The protocol is well thought out and has the potential to fill an important supportive care need.

We thank the Reviewer for these comments.

2. Additional detail on how BREATH SMART and BREATHE CDSS will overlap as these studies are running in parallel

BREATHE SMART and BREATHE CDSS are related but methodologically separate components of our broader program to improve the identification and management of chronic dyspnoea in general practice. BREATHE SMART is a proof-of-concept study evaluating the feasibility and acceptability of a digital self-screening pathway completed before the GP consultation. BREATHE CDSS is a separate study focused on developing and testing a clinical decision support system to guide GPs once a patient with potential chronic breathlessness has been identified. They form a complementary sequence: SMART supports earlier identification, while the CDSS supports structured diagnostic decision-making. This delineation and interconnectedness of the two studies has been explained in the 'Study design' section of the methods of the revised manuscript.

3. Including clarity on the eligibility criteria of who would be considered an eligible patient to include in the denominator would be useful. Would this be all-comers regardless of the ability to engage in screening due to language, visual deficits, etc.?

We agree that additional clarity on the in-scope population is important, and we have added the following points in the revised Methods section- that in-scope people

includes all eligible adult patients with a scheduled appointment, the screening rate reflects those who successfully receive, access, and complete the digital screening.

4. Although this study is proof of concept, it would be useful to identify the primary study outcome and any a priori definitions of how you would operationalize "success" for each outcome (e.g., what would be considered a successful proportion of eligible patients screened?)

We agree that distinguishing between primary and secondary outcomes, and clarifying how 'success' will be interpreted, strengthens the protocol. We have now updated the manuscript to explicitly identify the primary and secondary outcomes for the study.

As this is a proof-of-concept feasibility study, there are no established or clinically meaningful thresholds for what constitutes an acceptable level of uptake, workflow integration, or clinician acceptability for a digital pre-consultation screening pathway in Australian general practice. For this reason, we have not applied clinical performance benchmarks. Instead, we have added pragmatic, a priori feasibility indicators to guide interpretation of the findings and inform whether further refinement or a larger-scale evaluation is warranted.

5. Details on the randomization process would be useful. Any stratification of practices? Any additional details on how practices will be recruited to reduce bias?

Randomisation

Details of the randomisation process to BREATHE CDSS has been added to the 'Study design' section of the Methods. This additional text clarifies that practices are randomised to the CDSS at the practice level using a computer-generated allocation sequence prepared by an independent statistician who is not involved in recruitment or data collection and that allocation will occur only after a practice has formally consented to participate (ensuring concealment of assignment during the recruitment phase).

Stratification

Given the proof-of-concept nature of the study and the relatively small number of participating practices, we will not apply formal stratification. However, we will monitor key practice characteristics (e.g., size, rurality, socioeconomic context) to support interpretation of some of the outcomes measured, as well as the interpretations in the process evaluation.

Recruitment bias

The following information has been added to the 'Practice recruitment' section of the Methods, 'To reduce the potential for recruitment bias at the practice level, we will use a standardised recruitment approach. All practices will receive the same study information, and recruitment to the study will not be targeted based on practice performance, digital maturity, or patient demographics. We will document reasons for non-participation where provided, enabling transparent reporting of recruitment flow and potential sources of bias.'

6. Addressing some limitations of the design could be useful in the discussion. For example, the study appears to be operationalizing GPs' discussion of dyspnea screening results and management based on the number of dyspnea-related conditions reported at a site. Commenting on what could be missed with this operationalization could help provide further context on what this protocol can address or not. Similarly, only patients with a phone that opted in to receive SMS communications will be screened. This design choice, though absolutely understandable, has its own limitations that can be mentioned.

The Discussion has been revised to more clearly delineate what the study can and cannot address within its proof-of-concept scope.

First, we acknowledge that our operationalisation of GP engagement with screening results- using the number of dyspnoea-related conditions recorded at each site- captures only one dimension of how GPs may use the information. This approach does not allow us to observe the full breadth of clinical reasoning, nor does it capture instances where GPs may discuss breathlessness but not record a specific diagnostic code. As such, some nuances of GP-patient interaction and decision-making may be missed. We have clarified that this measure is intended as a pragmatic proxy for assessing whether the screening information prompts diagnostic consideration, rather than a comprehensive assessment of clinical management.

Second, we have added text acknowledging that only patients with a mobile phone who have opted in to receive SMS communications will be able to participate in the digital screening pathway. While this design choice reflects real-world implementation constraints and the current capabilities of the digital platform, it introduces limitations related to digital access, digital literacy, and patient preference. These factors may lead to under-representation of certain groups, including older adults, people with lower socioeconomic status, and those with limited English proficiency. We note that this may affect estimates of reach and uptake and should be considered when interpreting feasibility outcomes.

VERSION 2 - REVIEW

**Reviewer**	**1**
**Name**	**Lo, Stephen B**
**Affiliation**	**Ohio State University Health System**
**Date**	**06-May-2026**
**COI**	

Thank you for the opportunity to review this manuscript again. The authors have addressed my concerns well. I wish them the best on their research endeavors.
